# Cytosolic Glyceraldehyde-3-Phosphate Dehydrogenase Is Phosphorylated during Seed Development

**DOI:** 10.3389/fpls.2017.00522

**Published:** 2017-04-11

**Authors:** Claudia V. Piattoni, Danisa M. L. Ferrero, Ignacio Dellaferrera, Abelardo Vegetti, Alberto Á. Iglesias

**Affiliations:** ^1^Laboratorio de Enzimología Molecular, Instituto de Agrobiotecnología del Litoral (Consejo Nacional de Investigaciones Científicas y Técnicas – Universidad Nacional del Litoral) and Facultad de Bioquímica y Ciencias Biológicas (Universidad Nacional del Litoral), Centro Científico Tecnológico, Consejo Nacional de Investigaciones Científicas y Técnicas Santa FeSanta Fe, Argentina; ^2^Cultivos Extensivos, Facultad de Ciencias Agrarias, Universidad Nacional del Litoral, EsperanzaArgentina; ^3^Morfología Vegetal, Facultad de Ciencias Agrarias, Universidad Nacional del Litoral, EsperanzaArgentina

**Keywords:** glycolysis, phosphorylation, glyceraldehyde-3-phosphate, seeds, wheat, castor oil seed

## Abstract

Cytosolic glyceraldehyde-3-phosphate dehydrogenase (NAD-GAPDH) is involved in a critical energetic step of glycolysis and also has many important functions besides its enzymatic activity. The recombinant wheat NAD-GAPDH was phosphorylated *in vitro* at Ser205 by a SNF1-Related protein kinase 1 (SnRK1) from wheat heterotrophic (but not from photosynthetic) tissues. The S205D mutant enzyme (mimicking the phosphorylated form) exhibited a significant decrease in activity but similar affinity toward substrates. Immunodetection and activity assays showed that NAD-GAPDH is phosphorylated *in vivo*, the enzyme depicting different activity, abundance and phosphorylation profiles during development of seeds that mainly accumulate starch (wheat) or lipids (castor oil seed). NAD-GAPDH activity gradually increases along wheat seed development, but protein levels and phosphorylation status exhibited slight changes. Conversely, in castor oil seed, the activity slightly increased and total protein levels do not significantly change in the first half of seed development but both abruptly decreased in the second part of development, when triacylglycerol synthesis and storage begin. Interestingly, phospho-NAD-GAPDH levels reached a maximum when the seed switch their metabolism to mainly support synthesis and accumulation of carbon reserves. After this point the castor oil seed NAD-GAPDH protein levels and activity highly decreased, and the protein stability assays showed that the protein would be degraded by the proteasome. The results presented herein suggest that phosphorylation of NAD-GAPDH during seed development would have impact on the partitioning of triose-phosphate between different metabolic pathways and cell compartments to support the specific carbon, energy and reducing equivalent demands during synthesis of storage products.

## Introduction

Cytosolic glyceraldehyde-3-phosphate dehydrogenase (NAD-GAPDH, EC 1.2.1.12) is a homotetrameric glycolytic enzyme that catalyzes the phosphorylating-coupled oxidation of Ga3P: Ga3P + Pi + NAD^+^ ←→ 1,3-bisP-glycerate + NADH + H^+^. Then, the high energy intermediate 1,3-bisP-glycerate is metabolized by P-glycerate kinase (EC 2.7.2.3, PGK) to produce ATP and 3P-glycerate (3PGA): 1,3-bisP-glycerate + ADP ←→ 3PGA + ATP (Nelson and Cox, 2004). These two glycolytic steps are of relevance in plants. The process allows ATP synthesis by phosphorylation at the substrate level and is critical to establish the partition of triose-P [Ga3P and dihydroxyacetone-phosphate (DHAP)] between different cell compartments and metabolic pathways (Plaxton, 1996; Givan, 1999). NAD-GAPDH is an abundant protein that in plants was first highly purified and kinetically characterized from pea seed (Duggleby and Dennis, 1974a,b,c). Its catalytic role in glycolysis is based on a highly reactive catalytic cysteine that is often target of oxidative modifications that blocks its enzymatic activity and in turns trigger other moonlighting non-glycolytic roles (Sirover, 2012; Zaffagnini et al., 2013).

Glycolysis in plants presents alternatives to the classic route (occurring in most other organisms) allowing an adequate metabolic adaptation to the changing environmental conditions that they face owing their sessile life style (Givan, 1999). The Ga3P glycolytic metabolism is one step with alternatives in plants. The triose-P can be directly oxidized to 3PGA by the cytosolic non-phosphorylating Ga3P dehydrogenase (np-NADP-GAPDH, EC 1.2.1.9): Ga3P + NADP^+^ → 3PGA + NADPH + 2 H^+^, as an alternative to the steps mediated by NAD-GAPDH and PGK (Iglesias, 1990; Plaxton, 1996). The simultaneous occurrence of these two pathways establishes that Ga3P could be oxidized in the cytosol rendering energetic (ATP and NADH) or reducing (NADPH) power. The regulation of this branch point would effectively coordinate cellular production of energetic and reductive equivalents according to the transitory cell requirements. This view is supported by results showing that: (i) np-NADP-GAPDH is a target for regulation by post-translational modification (Bustos and Iglesias, 2002, 2003); and (ii) both GAPDHs are differentially regulated by redox mechanisms allowing modification of Ga3P partitioning to improve the production of NADPH by np-NADP-GAPDH under oxidizing intracellular cell imbalance (Piattoni et al., 2013). It was also shown that oxidized NAD-GAPDH (inactive), due to changes in the redox cell environment, participates in signal transduction cascades as a H_2_O_2_ sensor (Guo et al., 2012).

Previously, we reported that in wheat (*Triticum aestivum*) heterotrophic tissues np-NADP-GAPDH is phosphorylated at Ser404 by a SnRK1 (SNF1 related) protein kinase (Piattoni et al., 2011). The phosphorylated enzyme interacts with 14-3-3 regulatory proteins and consequently exhibits a lower activity and enhanced sensitivity to regulation by adenylates and inorganic pyrophosphate (Bustos and Iglesias, 2002, 2003). Further studies in wheat endosperm showed that nearly physiological concentrations of ribose-5-P (Rib5P), and to a lesser extent fructose-1,6-bisP (Fru1,6bisP) and 3PGA, inhibited SnRK1 activity, suggesting that the system would be modulated by the metabolic conditions occurring in the cell (Piattoni et al., 2011). As NAD-GAPDH is a counterpart of this glycolytic step, the question arises if it could also be a target of regulation by phosphorylation. Previous results agree with this view. In Arabidopsis cells, NAD-GAPDH was identified as partner of 14-3-3 regulatory proteins, together with nitrate reductase, sucrose-P synthase, glutamyl-tRNA synthase and a calcium dependent protein kinase (CDPK) (Cotelle et al., 2000). As well, different phospho-peptides are reported for NAD-GAPDHs from Arabidopsis in the PhosPhAT Database^1^ compiling phosphoproteomic studies carried on kinase mutants (Roitinger et al., 2015) and Arabidopsis ecotype Col 0 stimulated with auxin (Zhang et al., 2013), abscisic acid (Wang et al., 2013) or under osmotic stress (Xue et al., 2013). Even given these evidences, the post-translational modification of plant NAD-GAPDH by phosphorylation is far from complete.

In this work, we report that phosphorylation of NAD-GAPDH occurs in wheat and castor oil seeds. *In vitro* phosphorylation studies identified Ser205 as the residue being putatively modified by kinases extracted from heterotrophic (and not from photosynthetic) tissues of plants growing under natural conditions. Also, we describe that phosphorylation of NAD-GAPDH occurs *in vivo* with specific profiles of NAD-GAPDH activity, protein level and phosphorylation degree during seed development that, interestingly, were different in seeds accumulating starch (wheat) or lipids (castor oil seed).

## Materials and Methods

### Chemicals

Rabbit muscle aldolase, ATP, NAD, Fru1,6bisP, Rib5P, and 3PGA were purchased from Sigma Aldrich (St. Louis, MO, USA). All other reagents were of the highest quality available.

### NAD-GAPDH Production, Purification, and Activity Assay

Recombinant wheat NAD-GAPDH production, purification and assay for activity were performed as previously described (Piattoni et al., 2013). Briefly, recombinant NAD-GAPDH was obtained from *Escherichia coli* BL21Codon Plus^®^(DE3) RIL cells transformed with [pRSETB/*TagapC*], and the enzyme was purified to near homogeneity by metal affinity chromatography (HiTrap^TM^ Chelating HP, GE Healthcare). Enzyme activity assay (50 μl) contained (unless otherwise specified): 50 mM Tricine-NaOH pH 8.5, 1 mM NAD^+^, 10 mM sodium arsenate, 0.4 units aldolase, 1.2 mM Fru1,6bisP, and an adequate quantity of protein. NADH generation was monitored spectrophotometrically at 30°C and 340 nm. One unit (U) is defined as the amount of enzyme that catalyzes the formation of 1 μmol NADH per minute under the specified assay conditions.

Substrate saturation curves were performed by assaying activity at saturating level of the fixed substrate and different concentrations of the variable substrate. Values for the kinetic parameters for NAD^+^ and D-Ga3P were obtained fitting the experimental data to the generalized Hill equation by a non-linear least-square regression kinetics computer program (Brooks, 1992). All kinetic parameters are the mean of at least three independent set of saturation curves that were reproducible within ±10%.

### Seed Harvest

*Triticum aestivum* L. cv. Baguette 11, was cultivated in the experimental field of the Agronomic Sciences Faculty [Universidad Nacional del Litoral (UNL), Santa Fe, Argentina] from June 13th (sowing date) until December 20th in 2011 and 2013 [It should be noted that the experiment was done in the South hemisphere]. The wheat density was 4 × 10^6^ seedlings per hectare and plot dimensions were 10 × 30 m. Anthesis date was recorded when the first anther appears above the glumes in the central region of the spike. Samples were harvested at 3, 6, 10, 14, 17, and 27 days post-anthesis (DPA); and spikes frozen immediately in liquid nitrogen. Seed samples contained grains from the central part of the frozen spike between the fifth and tenth spikelet that were store at −80°C until analysis.

Castor (*Ricinus communis*) seeds were harvested from the field around the University Campus (Biochemistry and Biological Sciences Faculty, UNL, Santa Fe, Argentina) in 2011 and 2013. Castor seeds were reaping at various stages of development as previously described (Greenwood and Bewley, 1982). Collected seeds were dissected from the capsule, frozen immediately in liquid nitrogen, and stored at −80°C until analysis.

In both cases, non-frozen seeds were used for fresh weight, starch and triacylglycerol (TAG) determinations.

### Kinase Extraction and SnRK1 Partial Purification

Protein kinases were extracted as described previously (Piattoni et al., 2011). Briefly, wheat seeds collected after 20 DPA, or wheat leaves were ground to a fine powder in a mortar with liquid nitrogen and pestle. Following, kinase extraction buffer [50 mM HEPESKOH pH 7.5, 1 mM EDTA, 1 mM EGTA, 25 mM NaF, 0.1% (v/v) Triton X-100, 20% (v/v) glycerol, 10 mM MgCl_2_, 5 mM thiourea, 2 mM dithiothreitol (DTT), 2 mM phenylmethylsulfonyl fluoride (PMSF), 5 mM malate, and 1% (w/v) polyvinyl polypyrrolidone] was used to extract total proteins (Turner et al., 2005). The homogenate was centrifuged at 15,000 ×*g* for 15 min at 4°C and the supernatant immediately used for experimentation. For partial purification of wheat endosperm SnRK1, the kinase extract prepared from wheat seeds was purified as before strictly following the same methodology and using the same exhaustive controls we assayed before more than once to purify this kinase (Piattoni et al., 2011). The purification of the specific kinase was tested and followed by western blot using monoclonal antibodies against the αAMPK phospho-Thr172 (Cell Signaling Technology).

### *In vitro* Phosphorylation Assay

For *in vitro* phosphorylation of recombinant NAD-GAPDH, the purified enzyme (1 μg) was incubated under activity conditions determined for different plant protein kinases in previous works, essentially as already detailed (Piattoni et al., 2011). The seven phosphorylation conditions assayed were: I. Ca^2+^-independent SNF1-related protein kinase (WPK4): 20 mM Tris-HCl, pH 8.0, 1 mM EDTA, 0.1 mM PMSF, 20 mM MgCl_2_, and 100 mM ATP at 25°C (Ikeda et al., 1999). II. Ca^2+^-dependent salt overly sensitive-2 protein kinase (SOS2): 20 mM Tris-HCl, pH 7.2, 5 mM MgCl_2_, 0.5 mM CaCl_2_, 2 mM DTT, and 10 mM ATP at 30°C (Gong et al., 2002). III. Glycogen synthase kinase 3 (GSK3): 20 mM HEPES-KOH, pH 7.4, 15 mM MgCl_2_, 5 mM EGTA, 1 mM DTT, and 10 mM ATP at 25°C (Jonak et al., 2000). IV. Mitogen-activated protein kinase (MAPK): 25 mM Tris-MES, pH 7.5, 12 mM MgCl_2_, 2 mM EGTA, 1 mM DTT, and 25 mM ATP at 30°C (Lalle et al., 2005). V. Casein kinase II (CKII): 10 mM Tris-HCl, pH 7.4, 50 mM KCl, 10 mM MgCl_2_, and 100 mM ATP at 30°C (Jeong et al., 2004).. VI. TOUSLED nuclear protein kinase (Tsl): 50mM HEPES-KOH, pH 7.6, 150 mM NaCl, 10 mM MgCl_2_, 2 mM MnCl_2_, and 100 mM ATP at 25°C (Roe et al., 1997). VII. CDPK: 25 mM Tris-HCl, pH 7.5, 0.5 mM DTT, 10 mM MgCl_2_, 0.1 mM CaCl_2_, and 50 mM ATP at 30°C (Zhang et al., 2005).

Unless otherwise specified, reactions were performed in 20 μl with 2 μCi of [^32^P]-γ-ATP (Perkin Elmer) and were initiated by adding wheat endosperm or leaves extract as kinase resource, or partially purified SnRK1 from wheat endosperm. After reaction, the protein mixtures were resolved by electrophoresis under denatured conditions on discontinuous polyacrylamide gels (SDS-PAGE) according to Laemmli (1970). For detection of radioactivity incorporation, the gels were stained with Coomassie Brilliant Blue R-250, dried, and radioactivity incorporation detected by storing phospho-screen (GE Healthcare) exposure and scanning with the Typhoon^TM^ system (GE Healthcare).

### Site-Directed Mutagenesis

The QuikChange (Stratagene) site-directed mutagenesis method was used to introduce the S66A, S124A, S205A, and S205D mutations in the *TagapC* gene (Piattoni et al., 2013). In each case two complementary primers, with the mutation in the middle, were used plus 10 ng of the [pRSETB/*TagapC*] plasmid as the template. PCR conditions consist of 16 cycles at 95°C for 1 min, 55°C for 1 min, and 72°C for 10 min. *E. coli* Top 10 F’ (Invitrogen) cells were used for plasmid propagation and mutant selection. Desired mutations and the entire sequences of the GAPDHs were verified by double strand DNA sequencing.

### Seed Whole Protein Extraction

Frozen seeds were ground to a fine powder using a mortar and pestle frozen in liquid nitrogen. Then, freshly prepared cold extraction buffer was added (1 μl per mg of frozen powdered tissue). The composition of the extraction buffer was 10% (v/v) glycerol, 0.25% (w/v) BSA, 0.1% (v/v) Triton X-100, 50 mM Hepes/KOH pH 7.5, 10 mM MgCl_2_, 1 mM EDTA, 1 mM EGTA, 1 mM PMSF, and 1 mM DTT (Gibon et al., 2004). The mixture was incubated 20 min on ice with constant homogenization. The extracts were centrifuged 30 min at 4°C and 15,000 ×*g*. The supernatant was recovered and used immediately for determinations.

### Protein Quantification

Total protein concentration was determined by the modified Bradford assay (Bradford, 1976) using bovine serum albumin as a standard.

### Phosphorylated Protein Purification

Chromatography for purification of phosphorylated protein was achieved as previously described (Muszynska et al., 1986). Total proteins (1.2 mg) in 50 mM MES-NaOH pH 6.0 were loaded onto 100 μl of IDA-Fe^+3^ previously equilibrated with the same buffer. After 1 h incubation at room temperature and constant homogenization, non-adsorbed proteins were washed out twice with 2 ml of 50 mM Mes-NaOH, pH 6.0. For elution of the adsorbed proteins pH was increased in three steps. First, 5 volumes of 50 mM PIPES-HCl pH 7.2 were applied, then the adsorbed proteins were washed out with three volumes of 50 mM Tris-HCl pH 8.0 and finally phosphorylated proteins (tightly bound to the matrix) were eluted with two volumes of 50 mM of Tris-HCl pH 9.0. For phosphorylated protein analysis samples eluted at the different pH conditions were analyzed by SDS-PAGE.

### Immunoblotting

Western blotting was achieved by transferring the proteins resolved by SDS-PAGE to nitrocellulose membranes using a semi-dry blot system (BioRad). Immunodetection was carried out using either an NAD-GAPDH rabbit antiserum raised against the cytosolic recombinant wheat NAD-GAPDH, αAMPK phospho-Thr172 (Cell Signaling) or anti-actin from plants (AS13-2640, Agrisera). Primary antibodies were incubated with the membrane during 16 h at 15°C under agitation. The immunoblotting was revealed with the secondary antibody Alexa Fluor 647 goat anti-rabbit IgG (H+L; Invitrogen) and the fluorescence emissions were detected using a Typhoon 9400 scanner (GE Healthcare).

### Protein Dephosphorylation

For protein dephosphorylation we followed a protocol described in Bustos and Iglesias (2002). Proteins (600 μg) extracted from seeds were diluted in 50 mM Tris–HCl (pH 8.5), 1 mM EDTA, 10 mM MgCl_2_, 1.2 mM CaCl_2_, 20 mM 2-mercaptoethanol, 1 mM PMSF (Camoni et al., 2000) and 5 or 20 U of alkaline phosphatase (AlkPase, M182A, Promega). Samples were incubated at 37°C for 5 h, the reaction was stopped by the addition of 4X SDS-PAGE sample buffer. Samples were then analyzed by electrophoresis followed by western blotting.

### Protein Stability Assays

For protein stability, 60 μl of sample were diluted in 200 μl of Tris-HCl 50 mM pH 9.3, MgCl_2_ 1 mM and ZnCl_2_ 0.1 mM and incubated at 37°C for 0, 60, 120, and 240 min. Incubations were performed in the absence or presence of (2 μl) SETIII protease inhibitor cocktail EDTA-Free (Calbiochem). For proteasome inhibition 2 μl of MG132 proteasome inhibitor (Calbiochem) was added in the presence of SETIII. The reaction was stopped by the addition of 4X SDS-PAGE sample buffer. Samples were then analyzed by electrophoresis followed by western blotting.

### Starch Determination

For starch quantification in seeds, the protocols described by Reibach and Benedict (1982), and Baud and Graham (2006) were combined. Plant tissue (100 mg) ground in a mortar under liquid nitrogen was soaked with 500 μl of ethanol 95% at 4°C. Samples were centrifuged 10 min at 15,000 ×*g* and 4°C. Supernatant was discarded and the extraction repeated thrice to eliminate the soluble sugars. Pellets were dried at 60°C and weighed. Dried pellets were dissolved in 10 μl of distilled H_2_O per mg of extracted material and then the tubes hermetically closed were boiled for 1 h for starch solubilization. The tubes were centrifuged 10 min at 15,000 ×*g* and 4°C. The soluble fraction (20 μl) was added to 200 μl of 100 mM sodium acetate pH 4.5 plus 70 U of amyloglucosidase (1,4-α-D-glucan glucohydrolase) and incubated 16 h at 55–60°C for starch digestion. After centrifuging for 10 min at 15,000 ×*g* and 4°C, the resulting soluble sugars were quantified by an enzymatic colorimetric assay where the H_2_O_2_ produced by glucose oxidase is measured by a peroxidase coupled to a colorimetric compound. The reaction mixture (100 μl) consisted of 70 μl of the commercial reactive (10 kU/l glucose oxidase, 1 kU/l peroxidase, 0.5 mM 4-aminophenasone, 100 mM phosphate buffer pH 7.0, and 12 mM 4-hydroxibenzoates) and 30 μl of sample conveniently diluted. The reaction was developed during 10 min at 37°C and the product quantified at 492 nm. To correlate the quantity of starch and the concentration of soluble sugars, we constructed a calibration curve [glucose (mg/ml) versus starch (μg)] with a standard starch solution treated identically to the sample.

### Lipid Quantification

Lipid quantification was based on the method described by Folch et al. (1957). Plant tissue (200 μg) was ground to a fine powder in liquid nitrogen and lipids extracted with 0.2 ml of milliQ H_2_O and 3.8 ml of chloroform/methanol: 2/1 (v/v) solution. Tubes were hermetically closed and incubated 2 h at room temperature with gently mixing every 30 min. Samples were filtered on oil free filter paper previously washed with the extraction solution. Samples transferred to previously weighed tubes were vigorously mixed with 0.7 ml of 0.02 % CaCl_2_ solution in chloroform/methanol/H_2_O: 3/48/47 (v/v) and centrifuged 5 min at 3,000 ×*g* until phase separation. The upper phase was discarded and 0.7 ml of chloroform/methanol/H_2_O: 3/48/47 (v/v) were added. Samples were vigorously mixed, centrifuged as before until phase separation and the upper phase discarded by suction. After these washes, chloroform was evaporated at 45–50°C to obtain the lipids. After weighing, lipids were dissolved in isopropanol and TAGs quantified by an enzymatic colorimetric assay. TAGs were digested by lipase and the resulting glycerol phosphorylated to glycerol-3P by a specific kinase and then oxidized by a glycerol-3P oxidase producing H_2_O_2_. The last product was quantified through a peroxidase coupled to a colorimetric compound. The reaction mixture (505 μl) consisted of 500 μl of the commercial reactive (50 mM PIPES pH 7.5, 5 mM 4-clorophenol, 15 kU/l lipase, 1 kU/l glycerol kinase, 2.5 kU/l Gro3P oxidase, 0.44 kU/l peroxidase, 0.7 mM 4-aminophenasone and 0.18 mM ATP) and 5 μl of sample conveniently diluted. The reaction was incubated 10 min at 37°C and color quantified at 492 nm.

## Results

### Recombinant NAD-GAPDH Is Phosphorylated *In vitro* at Ser205

**FIGURE 1:**
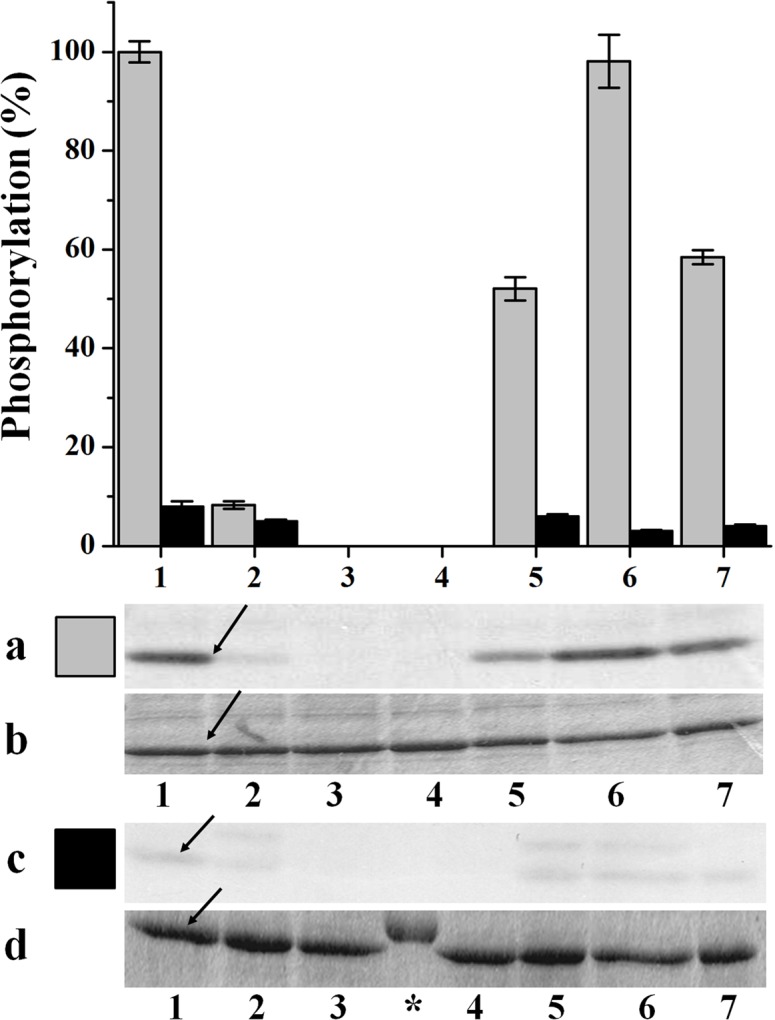
***In vitro* phosphorylation of recombinant *Tae*NAD-GAPDH by wheat seed and leaf extracts.** Schematic representation of NAD-GAPDH phosphorylation by different plant protein kinases extracted from seeds (gray) and leaves (black). NAD-GAPDH phosphorylation reactions resolved by SDS-PAGE and revealed by autoradiography **(a,c)** to visualize the NAD-GAPDH radioactivity incorporation from the [^32^P]ATP or stained with Coomassie Blue **(b,d)** to visualize all the proteins after phosphorylation by the plant kinases extracted from seeds and leaves, respectively. The NAD-GAPDH phosphorylation (%) was calculated from autoradiography using LabImage Version 2.7.0 (free edition) and all data are means of three independent experiments and reproducible with differences below ± 10%. Numbers indicate phosphorylation conditions for (1) WPK4, (2) SOS2, (3) GSK3, (4) MAPK, (5), CKII, (6) Tsl, and (7) CDPK. Arrows indicate the migration of NAD-GAPDH, of molecular mass in the range of 37 kDa. (^∗^) Molecular mass marker.

**FIGURE 2:**
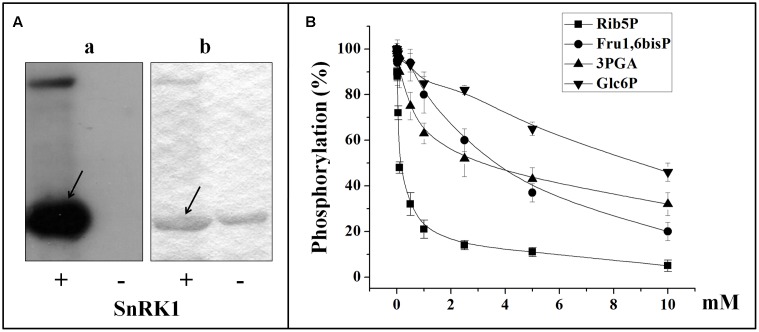
***In vitro* phosphorylation of recombinant *Tae*NAD-GAPDH by a SnRK1 purified from wheat seed. (A)** NAD-GAPDH radioactivity incorporation from the [^32^P]ATP after phosphorylation by the purified SnRK1 resolved by SDS-PAGE and revealed by storing phospho-screen exposure and scanning with the Typhoon^TM^ system (a) or stained with Coomassie Blue (b). Arrows indicate the migration of NAD-GAPDH. **(B)** Schematic representation of NAD-GAPDH phosphorylation by SnRK1 under the presence of different concentrations of Rib5P, Fru1,6bisP, 3PGA, or Glc6P. For schematic representations NAD-GAPDH phosphorylation (%) values were calculated from autoradiography using LabImage Version 2.7.0 (free edition) and all data are means of three independent experiments and reproducible with differences below ± 10%.

Different studies support that NAD-GAPDH would undergo post-translational modification by phosphorylation in plants (Cotelle et al., 2000; Wang et al., 2013; Xue et al., 2013; Zhang et al., 2013; Roitinger et al., 2015). Searching to gain knowledge in the subject, we sought to find a condition under which NAD-GAPDH could be phosphorylated *in vitro*, following the methodology previously employed for similar studies performed with np-NADP-GAPDH (Piattoni et al., 2011). Recombinant NAD-GAPDH from wheat (*Tae*NAD-GAPDH) was expressed and purified as described Piattoni et al. (2013). We assayed its capacity to be phosphorylated by incubating it with extracts from mature wheat seeds or leaves in the presence of [32P]ATP and under conditions previously determined to promote activity of different plant protein kinases (Roe et al., 1997; Ikeda et al., 1999; Jonak et al., 2000; Gong et al., 2002; Jeong et al., 2004; Lalle et al., 2005; Zhang et al., 2005). As illustrated by **Figure 1** (gray columns), NAD-GAPDH was phosphorylated by the seed extract in more than one condition, with the highest signal observed for that promoting WPK4 activity. We carried out experiments in parallel and under identical conditions, except that the extract was obtained from leaves instead of seeds. In this case, almost no phosphorylation of the recombinant enzyme was observed (**Figure 1**, black columns). These results suggest that the seed protein kinase that was able to phosphorylate NAD-GAPDH was either absent or inactive in the photosynthetic tissues analyzed. The absence or lack of activity of this kinase was not due to a general lack of protein kinase activity in our leaf extracts. Indeed, in control experiments performed with myelin basic protein, a general substrate for kinases, this substrate was equally phosphorylated in all incubation media performed with leaves or seeds extracts (data not shown). The latter suggests that under natural and normal growth conditions the phosphorylation of NAD-GAPDH in photosynthetic tissues would be negligible. It should be considered that in our studies we only determined phosphorylation in leaves harvested during day-time. Thus, the possibility of find differences in activity of the kinase in night-time leaves should not be discarded and is a subject for future research. For further studies, we partially purified a SnRK1 kinase (SNF1-related protein kinase) from wheat seeds strictly following the protocol assayed before (Piattoni et al., 2011) that was able to phosphorylate the recombinant NAD-GAPDH *in vitro* (**Figure 2A**). The partially purified protein kinase (**Figure 2B**) was inhibited by Rib5P and, to a lesser extent, by Fru1,6bisP and 3PGA. These results are similar to those previously reported for the characterization of the SnRK1 phosphorylating np-NADP-GAPDH (Piattoni et al., 2011), suggesting that the same kinase would be active to phosphorylate both GAPDHs. Although, the protocol we used to purify SnRK1 has been broadly employed to obtain plant protein kinases able to phosphorylate key enzymes of carbon metabolism and to study its regulation by glucose-6P and trehalose-6P (Toroser et al., 2000; Tang et al., 2003; Zhang et al., 2009), we cannot exclude the possibility that other kinases could be present in the partially purified extract. Consequently, the phosphorylation of GAPDH herein reported could be catalyzed by other protein kinases acting independently or in combination with SnRK1.

**FIGURE 3:**
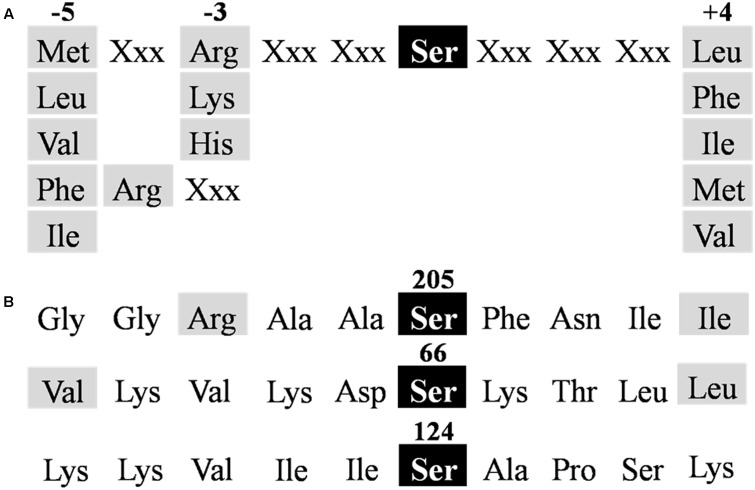
**Identification of *Tae*NAD-GAPDH phosphorylation motifs. (A)** Consensus sequence for SnRK1 targets (Halford et al., 2003). **(B)** NAD-GAPDH sequences identified as putative phosphorylation sites for SnRK1 in *Tae*NAD-GAPDH (NCBI N° ABQ81648.1). Residues required for recognition are highlighted in gray.

Protein phosphorylation generally occurs on Ser, Thr, or Tyr residues exposed to the solvent, accessible to interact with the kinase, and surrounded by characteristic amino acids (Boyer and Krebs, 1986). The protein motif for SnRK1 action should comprise the target Ser/Thr surrounded by hydrophobic residues at positions −5 and +4, plus at least one basic residue at position −3 or −4 (**Figure 3A**; Halford et al., 2003). Using *in silico* analysis of the *Tae*NAD-GAPDH protein sequence we identified (out of a total of 21 Ser and 22 Thr residues present in the enzyme) two SnRK1 recognition motifs (**Figure 3B**), surrounding the residues Ser205 and Ser66, the former having a better context. To study if these residues are involved in the phosphorylation of the enzyme, we performed site-directed mutagenesis to change these two Ser residues to Ala. We also produced an Ala mutant for the Ser124, as it has been previously reported as the probable site for the interaction of NAD-GAPDH with 14-3-3 regulatory proteins (Huber et al., 2002). We produced the recombinant mutant proteins following the same protocol used for the wild type enzyme. After purification, the enzymes were assayed for *in vitro* phosphorylation and kinetically characterized.

**FIGURE 4:**
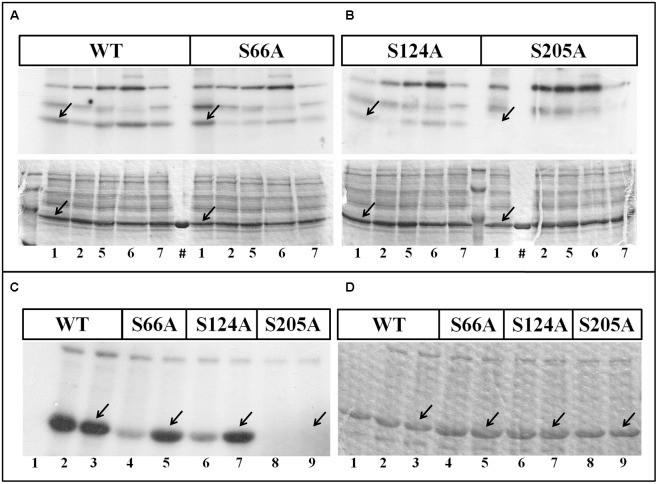
**Phosphorylation of *Tae*NAD-GAPDH versions. (A,B)** *In vitro* phosphorylation of NAD-GAPDH wild type and mutant S66A **(A)**, and NAD-GAPDH mutant S124A and S205A **(B)**, with plant extracts under kinases phosphorylation conditions for (1) WPK4, (2) SOS2, (5) CKII, (6) Tsl, and (7) CDPK. Incorporation of [^32^P]ATP was detected by autoradiography (upper image) of SDS-PAGE gel stained with Coomassie Blue (bottom image). (#) Recombinant proteins in the absence of the plant extract. **(C,D**) *In vitro* phosphorylation of all the NAD-GAPDH versions by the purified SnRK1 resolved by SDS-PAGE and revealed by storing phospho-screen exposure and scanning with the Typhoon^TM^ system **(C)** or stained with Coomassie Blue **(D)**. Numbers show the NAD-GAPDH versions: WT in the absence of SnRK1 (1); WT oxidized (2) or reduced (3) in the presence of SnRK1; S66A oxidized (4) or reduced (5) in the presence of SnRK1; S124A oxidized (6) or reduced (7) in the presence of SnRK1; S205A oxidized (8) or reduced (9) in the presence of SnRK1. Arrows indicate the migration of NAD-GAPDH. All the assays were also perform in the absence of any recombinant protein to identify the background phosphorylation.

Phosphorylation assays with wheat seed extract showed that S66A and S124A NAD-GAPDH mutants were phosphorylated in a similar way that the wild type enzyme, but the S205A mutant was recalcitrant to phosphorylation in any of the conditions assayed (**Figures 4A,B**). Additionally, we also phosphorylated *in vitro* all the NAD-GAPDH versions with the SnRK1 purified from wheat seeds (**Figures 4C,D**). We also tested the phosphorylation of the reduced and oxidized forms of the NAD-GAPDH versions, as redox regulation is also a post-translational modification affecting the enzyme activity and switching the protein to its non-glycolytic role (Zaffagnini et al., 2013). To reduce or oxidize the proteins we treated them as described before (Piattoni et al., 2013). As shown in **Figure 4C**, only the S205A (lines 8 and 9) mutant version was not phosphorylated by the SnRK1 purified from wheat seeds. The experimental data indicate that Ser205 is an *in vitro* phosphorylation site for NAD-GAPDH and also that SnRK1 would be the kinase involved in the phosphorylation reaction, thus supporting the *in silico* analysis showing the domain containing a Ser residue matching better with a SnRK1 phosphorylation motif. Moreover, results suggest that the different redox states undergone by NAD-GAPDH (Piattoni et al., 2013; Zaffagnini et al., 2013) has no major consequences for post-translational phosphorylation of the enzyme.

To further explore the kinetic consequences of the post-translational modification on the *Tae*NAD-GAPDH we produced and purified the S205D mutant enzyme, in which the replacement of the Ser by the Asp residue introduces a negative charge while maintaining size in order to mimic phosphorylation (Tarrant and Cole, 2009). The S205D and all the other mutant enzymes were characterized to obtain the kinetic parameters detailed in **Table 1**. As shown, in comparison with the wild type, S66A and S205A mutant enzymes exhibited similar affinity for both substrates but near half of the *V*_max_. However, *K*_m_ values for NAD^+^ and Ga3P determined for S124A were increased by 2.2-−and 2.8-fold, respectively, and the *V*_max_ decreased by 4.2-fold. Thus, S66A and S205A mutants exhibited ∼2-fold lower catalytic efficiency than the wild type enzyme in the use of both substrates. Interestingly, the NAD-GAPDH S205D mutant enzyme displayed a very low catalytic efficiency (∼330- and ∼410-fold lower for the use of NAD^+^ and Ga3P, respectively) with no significant changes in *K*_m_ values for substrates, but a 350-fold decrease in *V*_max_ when compared with the wild type. These results support that phosphorylation of NAD-GAPDH at Ser205 would impair enzyme catalysis.

**Table 1:** Kinetic characterization of *Tae*NAD-GAPDH Ser mutants^(#)^.

NAD-GAPDH type		WT	S66A	S124A	S205A	S205D
	*V*_max_ (U.mg^-1^)	37 ± 3	18.5 ± 0.5	9 ± 1	21 ± 1	0.11 ± 0.02
NAD^+^	*K*_m_ (μM)	126 ± 8	122 ± 6	299 ± 7	122 ± 4	130 ± 4
	*k*_cat_/*K*_m_ (M^-1^.s^-1^) ^∗∗^	1.66 × 10^5^	9.24 × 10^4^	1.83 × 10^4^	1.05 × 10^5^	5.15 × 10^2^
DGa3P	*K*_m_ (μM)	64 ± 3	74 ± 4	188 ± 6	98 ± 3	80 ± 2
	*k*_cat_/*K*_m_ (M^-1^.s^-1^) ^∗∗^	3.37 × 10^5^	1.52 × 10^5^	2.92 × 10^4^	1.3 × 10^5^	8.37 × 10^2^


^(#)^*Initial velocities were measured at variable concentrations of NAD^+^ (0.001–1.6 mM) or Ga3P (0.01–5 mM). NAD^+^ was fixed at 1.4 mM and Ga3P was maintained at 1.2 mM when the respective co-substrate was variable. Kinetic parameters were obtained from saturation plots as specified under Materials and Methods. ^∗∗^Catalytic constant (*k*_cat_) was calculated considering 36.6 kDa as the molecular mass of the monomeric enzyme.*

### NAD-GAPDH during Wheat Seed Development

**FIGURE 5:**
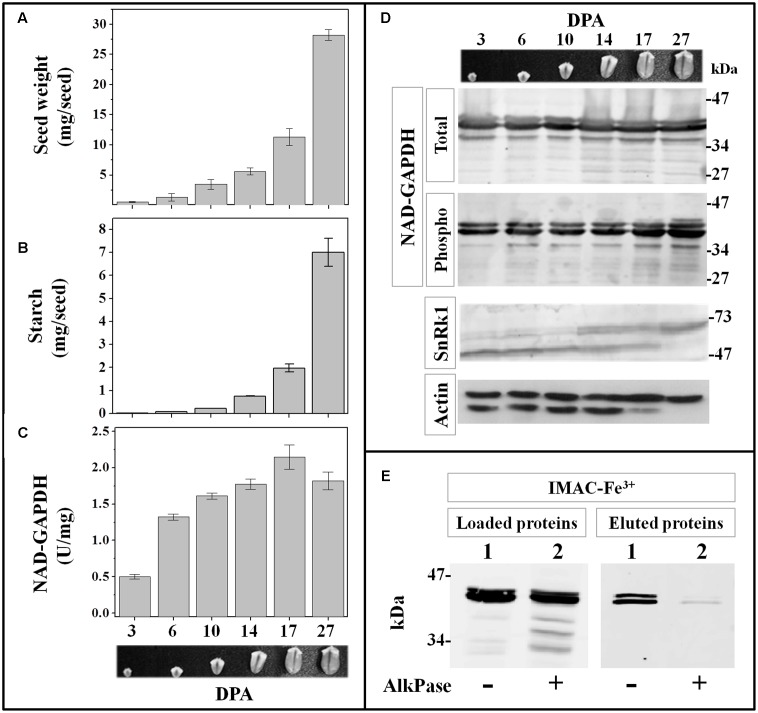
**NAD-GAPDH during wheat seed development.** Wheat seeds were collected at different DPA along development and the seed fresh weight **(A)** and starch content **(B)** determined immediately after sample collection. **(C)** NAD-GAPDH activity determined in total proteins extracted from each seed sample. **(D)** Immunoidentification of total NAD-GAPDH in whole protein extracts, phosphorylated NAD-GAPDH after phosphoprotein enrichment by IMAC-Fe^3+^ chromatography, SnRK1 and actin (used as a control) in whole protein extracts. **(E)** Immunoidentification of NAD-GAPDH in total wheat seed protein extracts at 27 DPA non-treated (lane 1) or treated (lane 2) with alkaline phosphatase before loading and after the IMAC-Fe^3+^ purification. In **A–C**, values are means of three independent assays determined by triplicate in two different biological samples. All immunoidentifications were performed in two different biological replicates and repeated independently twice in each replicate.

We found that *in vitro* phosphorylation of NAD-GAPDH could be catalyzed by SnRK1 protein kinases extracted from heterotrophic wheat tissues. Because this post-translational modification would negatively impact enzyme catalysis, we decided to explore activity, protein and phosphorylation profiles of NAD-GAPDH along seed development. The complete growth of wheat seeds is reached at 45 DPA^2^, being the respective phases of development: cell proliferation 0–10 DPA, accumulation of reserves 11–30 DPA, and seed maturation and desiccation among 30–45 DPA. For the analysis we collected wheat seeds from the experimental field at 6 different times: 3, 6, 10, 14, 17, and 27 DPA; and determined the fresh weight and the content of starch immediately after harvesting (**Figures 5A,B**, respectively). The profiles determined were similar to those established for wheat seed fresh weight (Briarty et al., 1979) and polysaccharide reserve accumulation (Dai, 2010). Starch synthesis initiates at 6 DPA, and progressively accumulates with an increase of ∼30-fold between 14 and 27 DPA, to constitute ∼25% of the seed fresh weight in the last sample analyzed.

**Figure 5** also illustrates the levels of NAD-GAPDH activity, protein phosphorylation status and total protein profiles determined in the extracts from each seed sample. NAD-GAPDH activity increased ∼3-fold after 3 DPA, and remained almost unchanged during the rest of seed development (**Figure 5C**). When the protein was detected with specific antibodies in the whole protein extracts (**Figure 5D**, NAD-GAPDH total), more than one polypeptide (∼34–47 kDa) were recognized, with minor changes in the protein abundance along development. When NAD-GAPDH was specifically detected in extracts enriched in phospho-proteins after IMAC-Fe^3+^ affinity chromatography, two major protein bands were detected in all samples through the end of the seed development (**Figure 5D**, NAD-GAPDH phospho). To confirm that the phospho-protein enrichment was related to the specific post-translational modification of the NAD-GAPDH, we assayed a de-phosphorylation treatment of the whole protein extract obtained from seeds at 27 DPA with alkaline phosphatase (AlkPase). After treatment, the extract was purified by IMAC-Fe^3+^ chromatography and the NAD-GAPDH specifically detected in the fractions loaded and eluted from the column (**Figure 5E**). As expected, after the treatment with AlkPase the IMAC-Fe^3+^ column retained significantly less amount of the enzyme, even though it is still being detected within the proteins loaded onto the column. This result confirms that purification of the phosphorylated NAD-GAPDH form in all the wheat seed samples analyzed is not due to an unspecific interaction with the IMAC-Fe^3+^ matrix.

In order to assess if SnRK1 is also present *in vivo* along development of wheat seeds, we specifically detected the kinase in the whole protein extracts using monoclonal antibodies against the αAMPK phospho-Thr172. This antibody recognizes α subunits of AMPK and it proved useful to detect SnRK1 in plants (Crozet et al., 2016), as the Thr172 and surrounding amino acids constitute the activation loop of the kinases related to AMPK and is highly conserved among plant species. As shown in **Figure 5D**, wheat seed extracts gave signal for SnRK1 all along development, but it seems that different isoforms of the kinase α-catalytic subunit would be expressed, as suggested elsewhere (Jain et al., 2008).

### NAD-GAPDH during Castor Oil Seed Development

**FIGURE 6:**
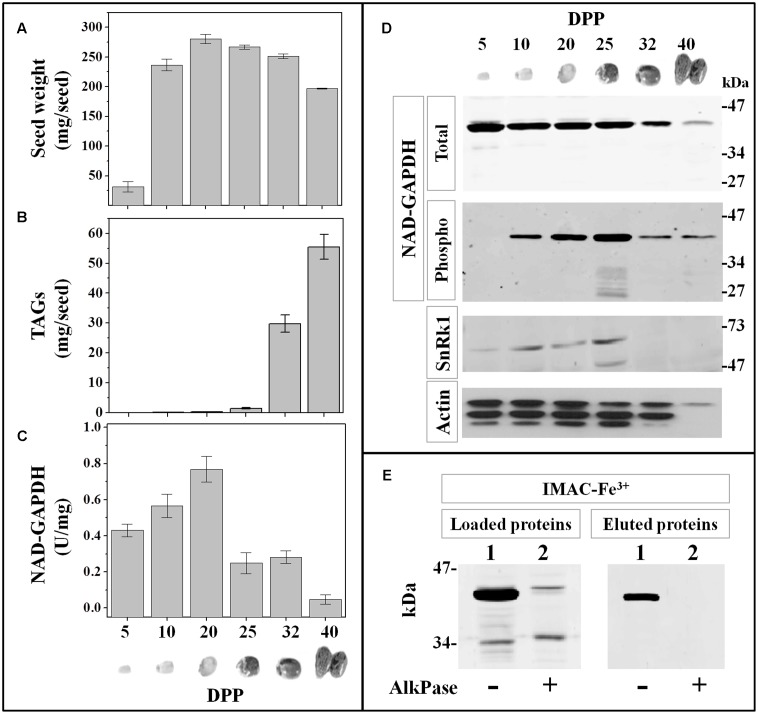
**NAD-GAPDH during castor oil seed development.** Castor seeds were collected at different DPP along development and the seed fresh weight **(A)** and TAG content **(B)** determined immediately after sample collection. **(C)** NAD-GAPDH activity determined in total proteins extracted from each seed sample. **(D)** Immunoidentification of: total NAD-GAPDH in whole protein extracts, phosphorylated NAD-GAPDH after phosphoprotein enrichment by IMAC-Fe^3+^ chromatography, SnRK1 and actin (used as a control) in whole protein extracts. **(E)** Immunoidentification of NAD-GAPDH in total castor seed protein extracts at 25 DPP non-treated (lane 1) or treated (lane 2) with alkaline phosphatase before loading and after the IMAC-Fe^3+^ purification. In **A–C**, values are means of three independent assays determined by triplicate in two different biological samples. All immunoidentifications were performed in two different biological replicates and repeated independently twice in each replicate.

Long-term storage of carbon in wheat seeds takes place in the endosperm and mainly as starch. Seeds like those of castor bean (*R. communis*) also store carbon in the endosperm, but in the form of TAGs instead of starch (Baud and Lepiniec, 2010). To compare NAD-GAPDH activity, protein profiles and phosphorylation between seeds storing TAGs or starch, we collected samples of castor seeds at different developmental ages. In castor oil seed development takes ∼60 days post-pollination (DPP) and the cell proliferation occurs between 0 and 20 DPP, the accumulation of reserves among 20–40 DPP, and the maturation and desiccation between 30 and 45 DPP (Canvin, 1963). The collected samples were classified in six groups according to the descriptive morphology given by Greenwood and Bewley (1982) as follows: 5, 10, 20, 25, 32, and 40 DPP. To better typify the developmental stages of each seed sample, we determined the fresh weight and TAG content immediately after harvesting (**Figures 6A,B**). The fresh weight profile agreed with the above referenced report, while the synthesis of TAGs initiated at 25 DPP and highly increased (∼40-fold) through 40 DPP, representing at this point ∼28% of the seed fresh weight. NAD-GAPDH profiles along castor seed development showed that the enzyme activity (**Figure 6C**) increased by 2-fold from 5 to 20 DPP, decreasing abruptly afterward (∼4-fold) at the point where the TAGs start to being accumulated and becoming almost undetectable at the end of the process. Regarding the protein profile, the specific antibodies recognized one polypeptide (around 34–47 kDa) remarkably diminishing in the last two seed sample analyzed (**Figure 6D**, NAD-GAPDH total). The immunodetection after phospho-protein enrichment by IMAC-Fe^3+^ (**Figure 6D**, NAD-GAPDH phospho) revealed that NAD-GAPDH also undergoes phosphorylation in castor seeds, with levels of the phospho-form exhibiting large variations along development. NAD-GAPDH phosphorylation seems to increase from 10 to 25 DPP and then decreased in agreement with the whole protein. Immunodetection of SnRK1 kinase with αAMPK phospho-Thr172 antibodies also shown that SnRK1 kinases are present from 5 to 25 DPP in castor developing seeds (**Figure 6D**, SnRK1); concurring with the profile detected for the phospho-NAD-GAPDH. To confirm that the phosphoprotein enrichment of NAD-GAPDH was specifically related with the phosphorylation status of the enzyme, we used AlkPase for de-phosphorylation and protein immunodetection before and after IMAC-Fe^3+^ purification in protein extracts from castor oil seeds at 25 DPP (**Figure 6E**). As a result, NAD-GAPDH was surprisingly neither detected in the IMAC-Fe^3+^ elution nor in the sample loaded in the column after AlkPase treatment, suggesting that the protein stability was affected. It is worth to note that controls determining levels of actin as a signal of same amount of protein loaded on the electrophoresis gels also exhibited a huge reduction of protein in samples of castor oil seeds at 40 DPP. This suggests that in this late stage of development, the seed may contain high levels of proteolytic enzymes which, together with the accumulation of oil could constitute an environment detrimental for the general stability of proteins. These instability conditions could start with more specificity (involving NAD-GAPDH) at earlier developmental periods and then could generalize to favor a tissue that finally mainly accumulates oils.

**FIGURE 7:**
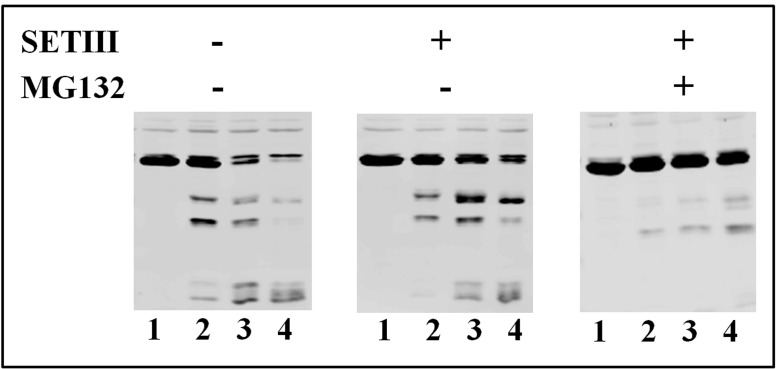
**Stability of NAD-GAPDH in extracts from castor seed at 25 DPP.** NAD-GAPDH was immunodetected in the respective extracts incubated at 37°C during 0 (lane 1), 60 (lane 2), 120 (lane 3), or 240 (lane 4) min; without further addition, in the presence of SETIII protease inhibitor cocktail alone and plus 10 μM of MG132 proteasome inhibitor.

Given the previous results showing that the NAD-GAPDH stability would be affected, we decided to perform protein stability assays in castor seed samples. For this purpose, extracts from castor seeds at 25 DPP were incubated from 0 to 240 min under conditions promoting protease activity in the absence or presence of the protease inhibitor cocktail SETIII alone or supplemented with 10 μM of the proteasome inhibitor MG132. As shown in **Figure 7**, immunodetection of the NAD-GAPDH in castor oil seed underwent rapid protein degradation in the absence or even in presence of SETIII, but the proteolytic process was significantly inhibited in the presence of MG132. These results support the view that NAD-GAPDH would be a target for degradation by the proteasome during seed development. Protein phosphorylation is a post-translational modification that can affect the activity, the structure, the localization and/or the stability of a protein (Nelson and Cox, 2004). The latter could be the case for NAD-GAPDH, but further studies are necessary to establish details on the mechanisms triggered by phosphorylation of the enzyme.

## Discussion

### Cytosolic NAD-GAPDH can be Phosphorylated by SnRK1

Cytosolic NAD-GAPDH is one of the most studied glycolytic enzymes and it has been found under post-translational regulation by redox mechanisms in many species (Guo et al., 2012; Piattoni et al., 2013; Zaffagnini et al., 2013). In this work, we performed an explorative assay in order to find *in vitro* phosphorylation conditions for the recombinant *Tae*NAD-GAPDH and we determined that the protein was phosphorylated with extracts from wheat seeds (but not from leaves) under phosphorylation conditions determined for SnRK, CKII, Tsl, and CDPK in previous works. For further studies, we partially purified a SnRK1 kinase from wheat seeds that exhibited the ability to phosphorylate *in vitro* the recombinant *Tae*NAD-GAPDH. An analysis of the NAD-GAPDH amino acidic sequence identified the Ser205 residue with the better chances to be the target of SnRK1 action, which was supported by the fact that the *Tae*NAD-GAPDH S205A mutant proved recalcitrant to phosphorylation. It is worth noting that NAD-GAPDH was found phosphorylated by AMPK in animal cells on a Ser residue equivalent to the Ser124 of the wheat enzyme (Chang et al., 2015). However, in our hands the absence of Ser124 had no impact on the protein phosphorylation *in vitro*, even though the replacement of the Ser124 by Ala markedly affected the catalytic properties of the enzyme. This is quite surprising, as phosphorylation of NAD-GAPDH by SnRK1 would be equivalent to the regulation mechanism found in animal cells. Interestingly, the alignment of the human, wheat and Arabidopsis NAD-GAPDH sequences showed that the −3 or −4 Arg residue critical for SnRK1 and AMPK action is missing in the plant enzymes around Ser124 (or equivalent) residue, while Ser205 (or equivalent) residue with an Arg at –3 only occurs in the plant enzymes. As it has been proposed that phosphorylation of Ser124 would trigger NAD-GAPDH interaction with 14-3-3 proteins (Huber et al., 2002), and given that the recombinant enzyme was also phosphorylated *in vitro* under other kinase conditions, the *in vivo* occurrence of more than one phosphorylation site should not be discarded. On the other hand, the fact that extracts from seeds but not those from leaves are able to phosphorylate NAD-GAPDH (and also np-NADP-GAPDH, see Piattoni et al., 2011) supports that SnRK1 and other protein kinases would have different kinetic/regulatory properties and play distinctive roles in photosynthetic or heterotrophic plant tissues.

**FIGURE 8:**
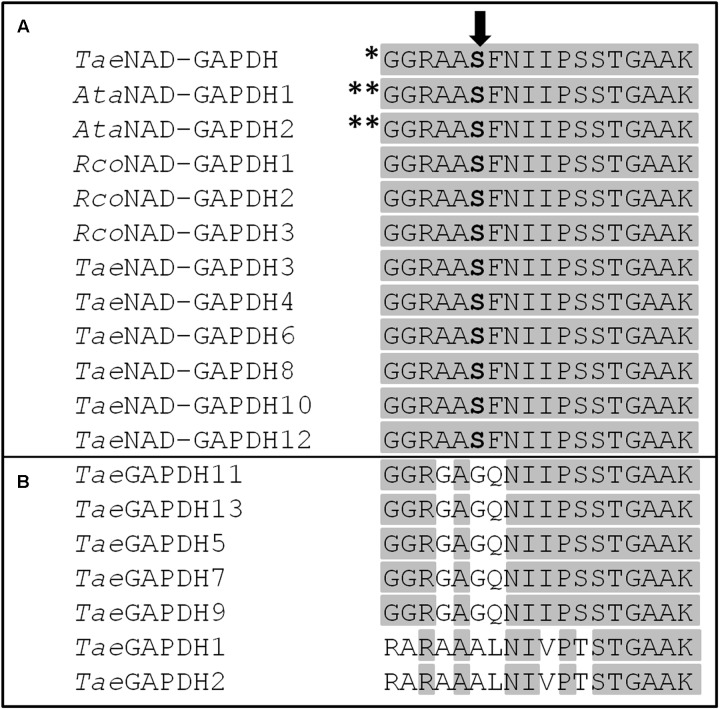
**Alignment of the phosphorylation motif studied in this work between different plant GAPDHs. (A)** Cytosolic NAD-GAPDHs and **(B)** plastidic TaeGAPDHs. *Ata*NAD-GAPDH 1 and *Ata*NAD-GAPDH 2 sequences are from TAIR corresponding to AT3g04120.1 and AT1g13440.1, respectively. *Tae*NAD-GAPDH and *Rco*NAD-GAPDH 1-3 sequences are from NCBI NABQ81648.1, XP_002509572.1, XP_002511235.1, and XP_002535536.1, respectively. *Tae*GAPDH 1-13 sequences were from Zeng et al. (2016). *Tae*GAPDH 1, 2, 5, 7, 9, 11, and 13 are isoforms found in different plastids. The Ser residue studied in this work is highlighted in bold. The amino acids conserved in all proteins are highlighted in gray. ^∗^Phosphorylation site identified *in vitro* in this work. ^∗∗^Phosphorylation sites identified *in vivo* for NAD-GAPDH in Arabidopsis with high number of items according to PhosPhAt database.

To assess how widely Ser205 or the equivalent residue in the phosphorylation motif is conserved between different plant species, we aligned the corresponding amino acids in all the sequences available for NAD-GAPDH in *Arabidopsis thaliana*, *T. aestivum* and *R. communis*. As shown in **Figure 8A**, the phosphorylation motif identified in this work is conserved in all the cytosolic NAD-GAPDH sequences analyzed. Meanwhile, the Ser residue is not conserved in plastidic *Tae*GAPDHs (**Figure 8B**), which supports that the phosphorylation site identified herein would be specific for cytosolic plant NAD-GAPDHs. It should be noted that the equivalent of wheat NAD-GAPDH Ser205 in both NAD-GAPDHs from Arabidopsis is the main phosphorylation site found *in vivo* according to PhosPhAT database^3^. Although this NAD-GAPDH phosphorylation site seems to represent a target that could have a physiological role in plants, the consequences of such a post-translational modification on the enzyme activity or stability were never studied before. In our work, we found that the S205D mutant (mimicking phosphorylation) exhibited a significant reduction in enzyme activity, thus suggesting that phosphorylation would have an inhibitory role on the enzyme. Previous reports point those enzymes like 3hydroxy-3-methylglutaryl-coenzyme A reductase (Sugden et al., 1999), nitrate reductase (Huber et al., 1992) and sucrose-P synthase (Huber et al., 1989) were also inactivated after being phosphorylated by SnRK1.

As we reported previously (Piattoni et al., 2011), the cytosolic np-NADP-GAPDH is also target of SnRK1 in wheat seed and both GAPDHs are phosphorylated *in vitro* at the same time by this kinase. Together, these results indicate that the cytosolic metabolism of triose-P would be under regulation by a protein kinase system that reduces the fate of Ga3P toward the action of both specific dehydrogenases. Nevertheless, as NAD-GAPDH is very abundant and there is no concrete evidence about that this reaction is regulated or displaced from equilibrium *in vivo*, its phosphorylation in plants could alternatively be a signal to shift its enzymatic role to other moonlighting non-glycolytic functions, as it was shown for the enzyme in animal cells (Chang et al., 2015). Besides, it is worth noting that the combined regulation of both cytosolic GAPDHs could not have a direct effect in the course of carbon from Ga3P to 3PGA in the glycolytic pathway, but it could affect generation of ATP plus NADH in detriment of NADPH or vice versa.

### On the NAD-GAPDH and Post-translational Modification during Seed Development

Seed development is genetically programmed and takes place in different phases. The pre-storage phase is highly mitotic and carbon fluxes are mainly used for the synthesis of cell compounds. During the next period a genetic reprogramming and metabolic adaptations switched the seed metabolism to the synthesis and accumulation of carbon reserves (Weber et al., 2005). Regulation of carbon metabolism is essential for seed growth as the metabolic changes will determine the partitioning of photosynthates, affecting the seed development and the composition of carbon reserves. Developmental changes in the activity, protein profiles and post-translational modification of NAD-GAPDH studied in this work contribute to the understanding of carbon metabolism and its regulation during seed development. Profiles of NAD-GAPDH activity and/or protein levels along seed development were different in wheat and castor seed. In wheat, the enzyme activity exhibited a progressive increase during development whereas in castor seed the activity only increased until TAG accumulation initiated, point at which NAD-GAPDH activity abruptly decreased at the end of development. The results on NAD-GAPDH changes in wheat seed fit well with the general glycolytic profile reported by Nadaud et al. (2010). These authors determined by proteomic analysis that in wheat endosperm glycolysis increases progressively through maturation until the beginning of the desiccation phase. Also, the immunodetection of two protein bands for cytosolic NAD-GAPDH in wheat is reminiscent of previous proteomic data (Vensel et al., 2005). Concerning the profiles for NAD-GAPDH in castor oil seed, data agree with the quantitative proteomic profile established during castor seed filling (Houston et al., 2009), as well as with a more recent proteomic analysis indicating that the relative protein abundance of the cytosolic NAD-GAPDH (UnitPort Accession number B9RBN8) decreases by 4-fold as seed develop (Nogueira et al., 2013). Even though the mutation of the Ser205 into an Asp clearly shows a significant decrease in GAPDH activity *in vitro*, we did not observed such a direct effect in enzyme assays determined in wheat extracts. These contradictory results could be explained considering the following different reasons: (i) the proportion of GAPDH phosphorylated specifically at Ser205 could be low *in vivo* in wheat; (ii) phosphorylation status could modulate the enzyme’s interaction with other components (i.e., 14-3-3 proteins) to be fully active/inactive; and/or (iii) the measurements of activity we performed could be also detecting the plastidic NAD-GAPDH that was shown to play a specific role in glycolytic energy production in non-green plastids (Petersen et al., 2003).

The comparative analysis after phosphoprotein enrichment showed that NAD-GAPDH could be controlled by phosphorylation in developing seeds, but with particular profiles in seeds accumulating starch or TAGs for long-term storage. In wheat, the phosphorylation remained almost unchanged during development; in contrast, in castor oil seed phosphorylation increased gradually during growth until TAGs start to be synthesized and accumulated. This constituted a break point where both, the protein and its activity, decreased to almost undetectable levels. SnRK1 immunodetection coincides with the profile found for phospho-NAD-GAPDH, supporting the *in vitro* phosphorylation of the recombinant NAD-GAPDH by SnRK1. The expression of different SnRK1 isoforms would occur in order to precisely regulate carbon allocation and metabolism during seed development, as was proposed in sorghum and maize (Jain et al., 2008). CDPK and SnRK1 are signals involved in the transition from the pre-storage to the storage/maturation phase (Weber et al., 2005), but the understanding of the targets phosphorylated is far from complete. Our results indicate that SnRK1 activity is related to NAD-GAPDH phosphorylation during seed. In addition we show that seeds storing different forms of carbon reserves have different SnRK1 and phospho-NAD-GAPDH profiles. It is tempting to speculate that these particular profiles would be related to the kind of reserves being accumulated, but further experiments with different seeds should be undertaken to prove or refute this hypothesis. Although SnRK1 is recognized as inhibiting growth under starvation, it has also been revealed to play a crucial role for seed filling and maturation (Crozet et al., 2014); the latter agreeing with our results on phosphorylation of NAD-GAPDH.

When NAD-GAPDH appears markedly phosphorylated, specifically at 27 DPP in castor oil seed, the protein seems to become less stable and subject to degradation. Even more, when the stability of NAD-GAPDH was assayed, proteolytic effect was significantly reduced by the addition of proteasome inhibitor reagent. It was previously shown that cytosolic pyruvate kinase is regulated by phosphorylation and proteolytic degradation during soybean seed development (Tang et al., 2003). Our results on NAD-GAPDH proteolysis are in agreement with that reported for this enzyme in sugar starved cell cultures of Arabidopsis, where it was demonstrated that the enzyme undergoes degradation, an effect also avoided by addition of MG132 (Cotelle et al., 2000).

Seeds are typically heterotrophic. Their supplies for carbon and energy mostly depend on the breakdown and oxidation of sucrose imported from photosynthetic tissues. In these cells, glycolysis is the main pathway using cleaved sucrose to provide the energy and building blocks for metabolism in different cell compartments. Glucose-6P, Ga3P and phospho*enol*pyruvate are the main glycolytic compounds being interexchanged between cytosol and organelles according to the cell requirements (Wang et al., 2015; Figueroa et al., 2016). At the beginning of seed development glycolysis fulfills the synthesis of cell components and respiration, but at later stages it supports carbon storage. At this point, the cell requires metabolic changes and the rearrangement of carbon precursor partitioning. Synthesis of starch and *de novo* production of fatty acids take place inside the plastid, and the relative proportion and localization of the reserves for long term storage in the seed (endosperm or embryo) will differ within plant species.

The analysis of NAD-GAPDH activity, protein and phosphorylation profiles in wheat and castor developing seeds, respectively, accumulating mainly starch or TAGs highlight some interesting clues. Even though these seeds are from different species and accumulate carbon in different ways, at the beginning (during the pre-storage phase) the carbon fate and partitioning would be similar, as both seeds need to supply the cell proliferation process. In fact, the profiles observed for NAD-GAPDH protein and activity during that phase (from 3 to 10 DPA in wheat and from 5 to 20 DPP in castor seed) looked similar in both plants; the NAD-GAPDH activity increases and the protein level remains with slight variations in both cases. Then, when the seed perform a metabolic adaptation to redirect carbon sources into reserves, the NAD-GAPDH profiles showed significant differences. In wheat, the NAD-GAPDH activity slightly increases but abruptly decreases in castor seed. Going ahead in development, NAD-GAPDH remains active and unchanged in wheat, but is almost undetectable in castor seed that are using carbon sources to supply the storage of TAGs. Precursors for fatty acids and TAGs synthesis are acetyl-CoA and glycerol-3P, both being synthesized from two glycolytic intermediates. Our results suggest that phosphorylation of NAD-GAPDH would switch the protein from its enzymatic role to other moonlighting non-glycolytic functions, as it was shown for the enzyme in animal cells (Chang et al., 2015). Phosphorylation would possibly target NAD-GAPDH for degradation, this impacting carbon partitioning just when TAGs start to be accumulated.

Oils and carbohydrates stored in seeds are not only essential reserves to support germination and early growth of plants, but they are also critical to humans and animals as major source of food (Vensel et al., 2005). Hence, research on carbon metabolism and its regulation during seed establishment is crucial for understanding the process and for future biotechnological applications. Some questions still remain. In particular, conclusive evidence connecting *in vivo* NAD-GAPDH phosphorylation at Ser205 in wheat and castor oil seed constitute the subject of future research. However, this work highlights that the post-translational modification process would play critical roles for determining carbon, energy and reducing power flux in plants during seed development.

## Author Contributions

CVP and AAI conceived and designed the project. CVP and DMLF generated experimental data and together with ID and AV collected and classified the seeds samples. CVP and AAI analyzed data and wrote the article.

## Conflict of Interest Statement

The authors declare that the research was conducted in the absence of any commercial or financial relationships that could be construed as a potential conflict of interest.

## Footnotes

1 http://phosphat.uni-hohenheim.de/db.html

2 http://bio-gromit.bio.bris.ac.uk/cerealgenomics/WheatBP/Documents/DOC_WheatBP.php

3 http://phosphat.uni-hohenheim.de/db.html
